# Climatic heterogeneity in meteorological associations with childhood acute respiratory symptoms across four South Asian countries

**DOI:** 10.7189/jogh.16.04239

**Published:** 2026-08-14

**Authors:** Yuran Lei, Wenqi Sha, Chensi Xu, Wanyi Kou, Yifan Zhang, Ruixin Guo, Ningrui Zhang, Yang Zhang, Zhenxing Wei, Zhenghui Wang

**Affiliations:** 1 Department of Otorhinolaryngology Head and Neck Surgery, Shaanxi Provincial Key Laboratory for Precision Diagnosis and Treatment of Otorhinolaryngology, The Second Affiliated Hospital of Xi'an Jiaotong University, Xi'an, China; 2 Department of Otolaryngology-Head and Neck Surgery, Luoyang Central Hospital Affiliated to Zhengzhou University, Luoyang, China

## Abstract

**Background:**

Childhood acute respiratory symptoms (ARSs) remain an important public health concern in South Asia, but meteorological associations may not be transferable across climatically diverse settings. Studies that rely on pooled regional estimates or single exposure metrics may therefore obscure meaningful heterogeneity. We examined whether associations of childhood ARSs with temperature and vapour pressure deficit (VPD) differed across macroclimatic regimes in four South Asian countries.

**Methods:**

We analysed Demographic and Health Survey data from India, Bangladesh, Nepal, and Timor-Leste, initially comprising 244,437 children aged <5 years aggregated into 33,407 survey cluster-month units. Childhood ARS was defined using a harmonised symptom-based measure based on caregiver-reported fast breathing and chest-related breathing difficulty during the preceding two weeks. Monthly temperature and VPD were assigned from ERA5 reanalysis data. We estimated the associations within country-macroclimate strata using mixed-effects binomial logistic regression models adjusted for demographic, socioeconomic, and household environmental covariates.

**Results:**

Meteorological associations with childhood ARS varied substantially across climatic regimes. Temperature-related associations were heterogeneous in both direction and magnitude across strata. By contrast, the clearest and most robust inverse association was observed for VPD in India’s arid macroclimate (odds ratio (OR) = 0.56; 95% confidence interval (CI) = 0.44, 0.71). In humid tropical settings, associations for both temperature and VPD were largely null. In India’s arid macroclimate, comparing the highest *vs* lowest VPD tertile corresponded to a risk difference of −1.28 percentage points (95% CI = −2.14, −0.71), equivalent to approximately 13 fewer ARS cases per 1000 children surveyed.

**Conclusions:**

Meteorological associations with childhood ARS were strongly context-dependent and should not be assumed to be uniform across climatic regimes. In this multicountry analysis, VPD showed the clearest association in India’s arid macroclimate, whereas temperature-related associations were more heterogeneous across settings. These findings highlight limitations of pooled regional interpretations and support climate-stratified approaches to meteorological risk assessment in child health surveillance.

**Keywords:**

childhood acute respiratory symptoms; South Asia; climatic heterogeneity; vapour pressure deficit; temperature; child health surveillance

Childhood acute respiratory symptoms (ARSs) remain an important cause of morbidity and healthcare utilisation in low- and middle-income countries, particularly in South Asia, where children are exposed to diverse climatic and social conditions [1,2]. Therefore, understanding how meteorological conditions relate to childhood respiratory risk is relevant to climate-sensitive child health surveillance and public health planning in the region [3]. However, although meteorological influences on respiratory outcomes are biologically plausible and increasingly studied, translating this evidence into locally useful guidance remains challenging [3,4].

A major challenge is that reported associations between meteorological exposures and respiratory outcomes are often inconsistent across settings [5]. Studies from tropical, temperate, and arid environments have produced heterogeneous, and sometimes opposing, estimates for temperature-related respiratory risk in children [6,7]. Rather than viewing these differences only as statistical noise or local anomalies, they may indicate a more fundamental problem for global health interpretation: exposure-response estimates derived from pooled or climatically dissimilar populations may not be readily transferable across settings.

Two common practices in climate-health research may contribute to this lack of transferability. First, many analyses pool data across regions with fundamentally different climatic structures, implicitly assuming that a single exposure-response relationship can be applied universally. Second, most studies rely on a single meteorological metric – typically ambient temperature – as a proxy for complex atmospheric conditions. While temperature is intuitively accessible, it does not fully capture the role of atmospheric moisture, which is increasingly recognised as relevant to respiratory pathogen dynamics and human physiological responses [4,8,9]. Consequently, pooled or temperature-centred estimates may be too general to support context-specific interpretation and, in some settings, may mislead public health prioritisation.

One potentially informative complement to temperature is vapour pressure deficit (VPD), an indicator of atmospheric dryness derived from temperature and humidity. Compared with temperature alone, moisture-related metrics may better reflect environmental conditions relevant to respiratory pathogen persistence, droplet behaviour, and mucosal defence [10–13]. However, VPD has rarely been evaluated alongside temperature within a framework that explicitly considers underlying climatic regimes. Its usefulness may therefore depend not only on the exposure metric itself, but also on the climatic context in which populations live.

To address these gaps, we examined associations between meteorological conditions and childhood ARS using a climate-stratified framework across four countries in the World Health Organization (WHO) South-East Asia Region. Instead of assuming a common regional exposure-response relationship, we stratified analyses by macroclimatic regime and compared temperature with VPD within each stratum. This design allowed us to assess whether meteorological associations differed meaningfully across climatic contexts and whether any observed differences could be translated into more interpretable, policy-relevant measures of population risk.

We aimed to assess whether meteorological associations with childhood ARS differed across macroclimatic regimes, to compare temperature and VPD within those strata, and to translate the clearest findings into interpretable public health terms. We sought to provide more context-aware evidence for climate-sensitive child health surveillance in climatically diverse settings.

## METHODS

### Study design and data source

We conducted a multicountry, climate-stratified analysis using data from Demographic and Health Surveys (DHSs) conducted from 2015 onward within the WHO South-East Asia Region. Among these, we included DHSs from India (2019–2021), Bangladesh (2017–2018), Nepal (2016–2017), and Timor-Leste (2016) because they had sufficiently comparable child respiratory symptom variables and compatible information for harmonised linkage with monthly meteorological exposures. The DHSs use nationally representative, multistage stratified cluster sampling and standardised questionnaires, allowing cross-country comparison of child health indicators. We reported this study in accordance with the STROBE guidelines (Checklist S1 in the **Online Supplementary Document**) [14].

### Study population and analytical units

The study population comprised all living children aged <5 years, defined as younger than 60 months, included in the Children’s Recode files from the four study countries. We excluded records with missing child age, survival status, survey cluster identifier, or interview date. Because meteorological exposures were assigned at the monthly level, individual observations were aggregated to the survey cluster-month level, which served as the primary analytical unit. For each cluster-month, the outcome was defined as the number of childhood ARS cases out of the total number of children observed. To improve analytical stability and reduce instability in binomial estimates based on very small denominators, we excluded cluster-month units with <10 children from the primary analyses. In the DHSs, a survey cluster corresponds to the primary sampling unit, typically a census enumeration area or comparable local sampling unit rather than a uniform administrative unit.

### Outcome definition

We operationalised childhood ARSs as a harmonised symptom-based outcome defined by the co-occurrence of caregiver-reported fast breathing and chest-related breathing difficulty during the two weeks preceding the survey. We did not include cough because its availability and coding were not fully comparable across the included survey datasets. This definition was selected to maximise cross-country comparability and specificity, but it likely captures a narrower and potentially more severe lower-respiratory symptom profile than broader community definitions of acute respiratory illness. Accordingly, the outcome should be interpreted as a narrower symptom-based construct rather than as representing the full spectrum of childhood ARSs, particularly mild or predominantly upper respiratory infections.

### Meteorological exposures

Monthly meteorological exposures were obtained from the ERA5 reanalysis dataset and included two-metre air temperature and dewpoint temperature. We linked survey cluster locations to gridded meteorological fields using cluster coordinates and bilinear interpolation. We included VPD, derived from air temperature and dewpoint temperature using standard physical equations, as a moisture-related indicator of atmospheric dryness. We also analysed the monthly mean two-metre air temperature in parallel as the conventional comparator. Pairwise correlations among temperature, absolute humidity, and VPD are presented in Table S4 in the **Online Supplementary Document**.

### Climate stratification

We assigned each survey cluster a Köppen–Geiger climate class using a 0.1°C global climate classification map. To ensure adequate sample size and stable estimation within strata, we collapsed detailed Köppen–Geiger classes into four macroclimate categories: ‘tropical’, ‘arid’, ‘temperate’, and ‘cold’. Polar classes, when present, were merged into the ‘cold’ category. We stratified primary analyses by country and macroclimate. For the main-text analyses, we further restricted inference to country-macroclimate strata with at least 150 cluster-month observations, 30 clusters, and 50 childhood ARS cases to reduce instability from sparse strata.

### Covariates

Covariates were summarised at the cluster-month level and included the proportion of male children, mean child age in months, proportion urban, mean household wealth index, mean maternal education level, and the proportion of households using solid fuels for cooking. We included these variables to account for demographic, socioeconomic, and household environmental conditions that might confound associations between meteorological exposures and childhood ARSs.

### Statistical analysis

Within each country-macroclimate stratum, we fitted mixed-effects binomial logistic regression models to cluster-month counts, specifying the number of childhood ARS cases out of the total number of children observed in each cluster-month. All models included a random intercept for survey cluster and fixed effects for survey year and calendar month. Cluster-month covariates included the proportion of male children, mean child age, proportion urban, mean household wealth, mean maternal education, and the proportion of households using solid fuels. Continuous exposures and covariates were standardised as z scores before modelling.

Primary analyses used separate single-exposure models for temperature and VPD. Results are reported as odds ratios (ORs) with 95% confidence intervals (CIs). Primary analyses were unweighted. To assess robustness to the DHS complex sampling design, we conducted probability-weighted individual-level analyses in India, the largest contributing country, using survey sampling weights. When weighted mixed-effects estimation was unstable, we used weighted logistic regression with cluster-robust standard errors. We also conducted secondary bivariable models including both temperature and VPD (**Online Supplementary Document**).

We used *R*, version 4.5.0 (R Core Team, Vienna, Austria) for all analyses.

### Supplementary and sensitivity analyses

We explored nonlinear exposure-response relationships for VPD in strata with large sample sizes using restricted cubic splines with three degrees of freedom. To support policy-relevant interpretation, adjusted absolute risks and risk differences were estimated via marginal standardisation for India’s arid macroclimate. Robustness checks included testing interactions between VPD and household wealth, comparing alternative methods for handling missing solid-fuel data, evaluating alternative exposure lag structures where feasible, and assessing the contribution of cluster-level random effects using variance components and information criteria (Table S2 in the **Online Supplementary Document**). We also conducted exploratory analyses of PM2.5 and meteorological variables in India’s arid macroclimate (Table S5 in the **Online Supplementary Document**).

## RESULTS

### Study population and analytical units

We included DHS data from India, Bangladesh, Nepal, and Timor-Leste (**Table 1**). The primary analytical unit was the survey cluster-month. The initial aggregated dataset comprised 33,407 cluster-month units representing 244,437 children aged <5 years, among whom 6638 ARS episodes were identified. After excluding cluster-month units with <10 children, 8821 cluster-month units representing 118,884 children remained. After a small number of additional exclusions due to final analytical filters, the final analytical dataset comprised 8740 cluster-month observations representing 117,610 children, including 3391 ARS cases.

**Table 1:** Survey periods and final analytical sample characteristics

Country	Survey period	Cluster-month units, n	Children <5 years, n	ARS cases, n	Prevalence, %
India	June 2019 to May 2021	7,710	101,937	2,973	2.9
Bangladesh	October 2017 to March 2018	465	6,723	205	3.0
Nepal	November 2016 to June 2017	246	3,800	104	2.7
Timor-Leste	September 2016 to December 2016	319	5,150	109	2.1

ARS – acute respiratory symptom

### Geographic and climatic coverage

**Figure 1:**
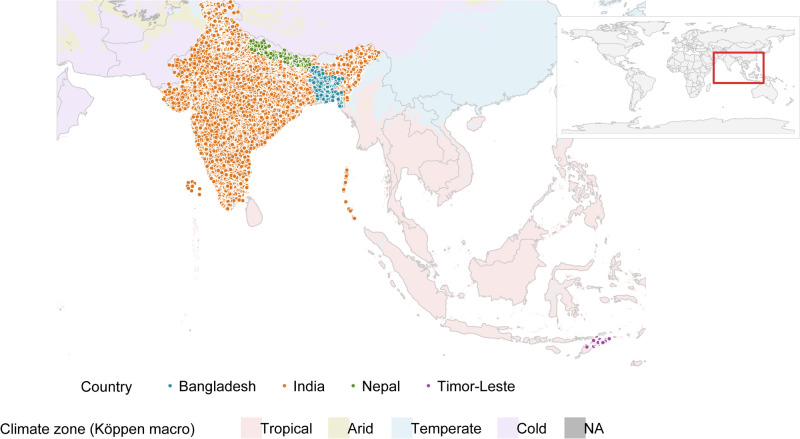
Geographic and climatic coverage of DHS clusters across four countries in the WHO South-East Asia Region. Cluster locations are overlaid on Köppen–Geiger macroclimate classifications. DHS – Demographic and Health Survey, NA – not available, WHO – World Health Organization.

Survey clusters were widely distributed across the four study countries, but climatic coverage differed substantially by country (**Figure 1**). India included tropical, arid, temperate, and cold macroclimates, indicating marked within-country climatic heterogeneity. Bangladesh was predominantly tropical with limited temperate representation, Nepal included temperate and cold macroclimates, and Timor-Leste was represented only by tropical clusters. These patterns confirmed that the study population was distributed across markedly different climatic settings, supporting climate-stratified analyses rather than a single pooled regional exposure-response pattern.

### Country- and macroclimate-specific associations

**Figure 2:**
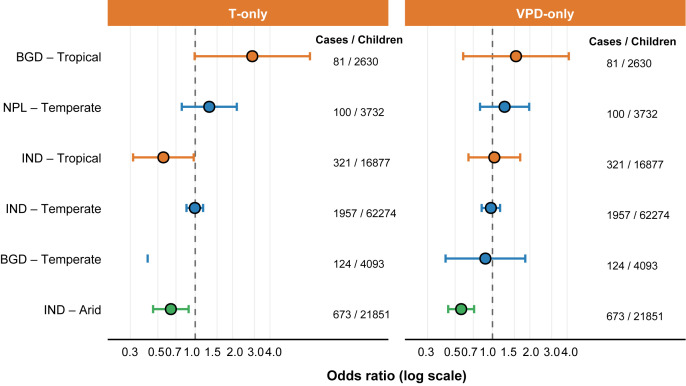
Country- and macroclimate-specific associations of T and VPD with childhood ARSs. **Panel A.** T-only. **Panel B.** VPD-only. Estimates were derived from the primary unweighted mixed-effects models in strata retained for the main-text analyses. ARS – acute respiratory symptom, BGD – Bangladesh, IND – India, NPL – Nepal, T – temperature, VPD – vapour pressure deficit.

Country- and macroclimate-specific associations of childhood ARSs with temperature and VPD differed substantially across climatic strata, with variation in both effect magnitude and direction (**Figure 2**, Panels A and B; Table S1 in the **Online Supplementary Document**).

Temperature-related associations varied considerably across macroclimates. In some strata, higher temperature was associated with lower ARS risk, whereas in others estimates were close to the null or imprecise. For example, in India’s arid macroclimate, higher temperature was associated with lower ARS risk (OR = 0.64; 95% CI = 0.46, 0.88), but temperature-related estimates were less consistent across tropical, temperate, and cold settings.

By contrast, the clearest and most robust pattern for VPD was observed in India’s arid macroclimate, where higher VPD was inversely associated with childhood ARSs (OR = 0.56; 95% CI = 0.44, 0.71). Across humid tropical settings, VPD-related estimates were generally close to the null, whereas estimates in smaller or colder strata were more variable and less precise.

Overall, these results indicate that meteorological associations with childhood ARSs differed importantly across climatic regimes rather than supporting a single pooled regional pattern. Pre-specified sensitivity analyses were directionally consistent for the main VPD finding in India’s arid macroclimate, whereas estimates in other strata were less stable and generally had wider CIs. Weighted individual-level analyses in India were broadly supportive but were not directly equivalent to the primary cluster-month models (Table S3 in the **Online Supplementary Document**).

### Absolute risk translation in India’s arid macroclimate

**Figure 3:**
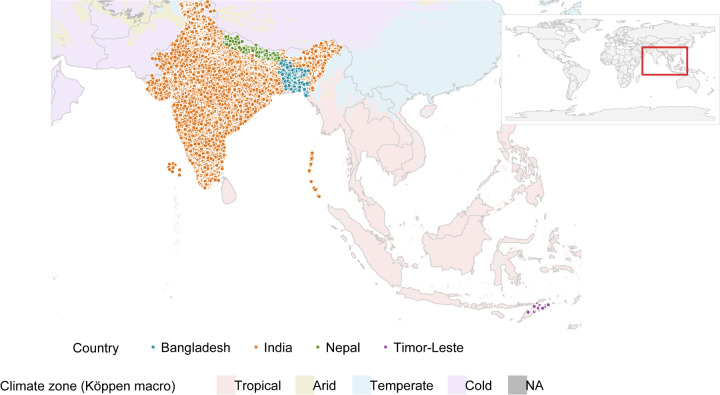
Absolute risk translation of the VPD association in India’s arid macroclimate. **Panel A.** Adjusted predicted probability of ARS. Shaded areas indicate 95% confidence intervals. **Panel B.** Adjusted ARS risk. Bars represent adjusted risks, and error bars denote 95% confidence intervals. ARS – acute respiratory symptom, IND – India, VPD – vapour pressure deficit.

Because the clearest inverse association for VPD was observed in India’s arid macroclimate, we translated this relative association into adjusted absolute risk measures to support population-level interpretation (**Figure 3**, Panels A and B).

Adjusted predicted ARS risk declined across increasing VPD tertiles in India’s arid macroclimate. The estimated ARS risk was 2.03% (95% CI = 1.68, 2.96) in the lowest tertile, 1.50% (95% CI = 1.36, 2.06) in the middle tertile, and 0.75% (95% CI = 0.64, 1.17) in the highest tertile. The absolute risk difference comparing the highest *vs* lowest tertile was −1.28 percentage points (95% CI = −2.14, −0.71), corresponding to approximately 13 fewer ARS cases per 1000 children surveyed.

Pre-specified sensitivity analyses supported the robustness of the inverse VPD association observed in India’s arid macroclimate. These analyses included probability-weighted individual-level models in India, bivariable models including both temperature and VPD, alternative handling of missing solid-fuel data, alternative temporal and lag specifications where feasible, and assessments of cluster-level random effects and VPD-household wealth interaction. Across these analyses, the VPD estimates generally remained inverse, although the magnitude and precision varied, indicating directional consistency with the primary model.

## DISCUSSION

In this multicountry analysis, we found that associations between meteorological exposures and childhood ARSs differed importantly across climatic regimes. Although both temperature and VPD have been examined in previous studies [2,6], we observed substantial heterogeneity in both the direction and magnitude of estimated associations across country-macroclimate strata. Temperature-based associations varied markedly across tropical, temperate, and arid settings, limiting their transferability across climatic contexts. By contrast, VPD showed the clearest and most robust inverse association in India’s arid macroclimate. Taken together, these findings challenge the implicit assumption that a single meteorological metric – or a pooled regional estimate – can adequately characterise climate-health relationships across diverse environments [15,16].

Beyond the specific comparison between temperature and VPD, our findings point to a broader issue in environmental health research: the interpretation of meteorological exposures depends strongly on climatic context [15,16]. The usefulness and interpretation of exposure metrics appear to depend on the climatic regimes in which populations live [17]. A single pooled effect estimate may therefore be not only imprecise but actively misleading when applied to regions with distinct climate structures [18]. Moreover, the most informative exposure metric – whether VPD, temperature, or another indicator – may itself be context-dependent rather than universally transferable. Recognising and operationalising this principle may help reconcile seemingly contradictory findings in the existing literature and improve the credibility of climate-health inference across heterogeneous settings [15,16].

The inverse association between VPD and childhood ARS in India’s arid macroclimate provides the clearest example of this context dependence. At first glance, this pattern may seem counterintuitive, given concerns that atmospheric dryness can impair mucosal defences or exacerbate respiratory irritation [12]. However, a higher VPD reflects greater atmospheric dryness and may plausibly be related to changes in pathogen persistence, droplet dynamics, household ventilation, or behavioural exposure patterns during the driest periods. These mechanisms cannot be distinguished in symptom-based observational data, but the observed pattern suggests that atmospheric moisture may represent an important component of respiratory risk in this arid setting [4].

Translating relative associations into absolute risk measures makes the findings easier to interpret from a public health perspective [19]. In India’s arid macroclimate, higher VPD was associated with about 13 fewer ARS cases per 1000 children surveyed when the highest exposure tertile was compared with the lowest. This translation provides a more interpretable sense of the potential public health relevance of the observed association [20]. Such quantification supports consideration of moisture-related indicators alongside temperature in climate-sensitive surveillance and risk assessment, particularly in arid settings [21].

Although our models adjusted for household wealth and solid-fuel use, the relevance of ambient atmospheric conditions may be greater in settings where households have limited capacity to modify indoor microclimates [22]. In many low-resource households, especially in arid regions, structural constraints on housing, ventilation, and cooling may amplify the health relevance of ambient atmospheric conditions [23]. This highlights the importance of public health strategies that take both environmental context and underlying social vulnerability into account [24]. From an environmental health perspective, these findings also suggest that incorporating moisture-related indicators, such as VPD, may improve the interpretation of meteorological risk signals in arid settings, whereas temperature alone may be insufficient to characterise environmentally relevant respiratory risks across climatically diverse populations [25–27]. In contrast, in humid tropical settings – where associations with both temperature and VPD were largely null – other environmental or social determinants, including air pollution, crowding, or seasonal infection dynamics, may warrant greater emphasis. Such climate-stratified targeting may improve the relevance of adaptation strategies without assuming uniform meteorological drivers across the region [25]. Future research should prioritise better temporal alignment between symptom timing and exposure assignment, improved characterisation of household environmental conditions, and replication of the arid-zone VPD finding in other settings.

This study has several limitations. First, the outcome was based on caregiver-reported symptoms rather than clinically confirmed diagnoses. In addition, because cough was not comparably available across all included DHSs, our harmonised definition used fast breathing together with chest-related breathing difficulty, which likely captures a narrower and potentially more severe lower-respiratory symptom profile than broader community definitions of acute respiratory illness. As a result, our findings may not generalise to mild upper respiratory tract infections or to the full spectrum of ARS presentations in children. Second, the DHS data are observational and cross-sectional in timing, which limits causal interpretation. Third, meteorological exposures were assigned at the monthly cluster level, whereas ARS referred to symptoms during the preceding two weeks, introducing temporal misalignment. This likely resulted in some non-differential exposure misclassification and may have attenuated associations towards the null, although more complex bias cannot be excluded. Fourth, DHS cluster coordinates are geographically displaced to protect confidentiality, which may have introduced additional exposure misclassification. Furthermore, we conducted the primary analyses at the cluster-month level, which improves comparability between meteorological exposures and survey timing but limits individual-level interpretation. The primary analyses were also unweighted, although weighted sensitivity analyses in India were broadly supportive of the main findings. Additionally, some country-macroclimate strata were small, leading to imprecise estimates in selected settings. Finally, residual confounding from unmeasured environmental, behavioural, infectious, or healthcare access factors cannot be excluded. Solid-fuel use was used as a pragmatic household-level proxy for indoor environmental conditions, but it does not fully capture variation in stove type, cooking location, ventilation, or child proximity to cooking activities. Exclusion of cluster-month units with <10 children may also have introduced selection bias by preferentially retaining larger sampled units, although we applied this restriction to improve the stability of binomial estimates.

## CONCLUSIONS

Our findings suggest that meteorological associations with this symptom-defined measure of childhood ARSs are context-dependent and should not be assumed to be uniform across climatic regimes. In this multicountry analysis, VPD showed the clearest and most robust association in India’s arid macroclimate, whereas temperature-related associations were more heterogeneous across settings. These results support the use of climate-stratified approaches when interpreting meteorological exposures for environmental health and child health surveillance.

## Additional material


Online Supplementary Document

## Data Availability

**Data availability:** The DHS data analysed in this study are available from the DHS Program, subject to registration and data access approval. We derived meteorological variables from the ERA5 reanalysis dataset produced by the European Centre for Medium-Range Weather Forecasts and accessed through the Copernicus Climate Data Store. Climate classification data were based on the Köppen–Geiger climate classification. The processed aggregate datasets and analytical code used to generate the results are available from the corresponding author upon reasonable request, subject to relevant data-use conditions.
