# Supplementary material for: Climatic heterogeneity in meteorological associations with childhood acute respiratory symptoms across four South Asian countries
Source: J Glob Health. 2026 Aug 14;16:04239. doi: 10.7189/jogh.16.04239 (PMC13474056; doi:10.7189/jogh.16.04239)
Supplement: Supplementary file 1 [file jogh-16-04239-s001.pdf]

Supplement to: Lei Y, Sha W, Xu C, Kou W, Zhang Yi, Guo R, Zhang N, Zhang Ya, Wei Z, Wang Z. Climatic heterogeneity in meteorological associations with childhood acute respiratory symptoms across four South Asian countries. J Glob Health. 2026;16:04239.

Supplementary Appendix

Supplementary Methods

S1. Survey time harmonisation

Survey timing was harmonised across countries using the DHS century month code (CMC; v008/v008a), which encodes the interview date as months since January 1900. CMC values were converted to a calendar year and month for analysis.

In Nepal, when valid CMC values were unavailable, survey dates recorded in the Bikram Samvat calendar were converted to Gregorian year and month for harmonisation. All survey dates were expressed in a uniform "YYYY-MM" format to ensure temporal alignment with monthly meteorological exposures.

S2. Meteorological derivation

Monthly meteorological data were obtained from the ERA5 reanalysis dataset, including two-metre air temperature and two-metre dewpoint temperature. Cluster-level exposures were assigned via bilinear interpolation using cluster centroid coordinates.

Vapour pressure deficit (VPD) was calculated from temperature and dewpoint temperature using standard thermodynamic equations. Absolute humidity was computed for supplementary analyses but was not included in the primary models.

S3. Climate classification and analytical strata

Primary analyses were stratified by country and Köppen–Geiger macroclimate category. Climate classifications were derived from a 0.1° global map and assigned based on cluster location. Detailed classes were collapsed into four macroclimates (Tropical, Arid, Temperate, and Cold) to ensure adequate sample size. Stratification was specified a priori and not modified based on statistical

significance. For the main-text analyses, strata with fewer than 150 cluster-month observations, fewer than 50 ARS cases, or fewer than 30 clusters were considered too sparse for stable primary inference.

S4. Primary model specification

The primary analyses reported in the main text were conducted at the survey cluster-calendar month level within each country-macroclimate stratum. For each stratum, we fitted mixed-effects binomial logistic regression models to cluster-month counts, specifying the number of ARS cases out of the number of children observed in each cluster-month. Models included a random intercept for survey cluster and fixed effects for survey year and calendar month, and adjusted for cluster-month covariates summarising the proportion of male children, mean child age, proportion urban, mean household wealth, mean maternal education, and proportion of households using solid fuels. Continuous exposures and covariates were standardised as z scores. Primary analyses were unweighted.

S5. Supplementary analyses

A series of supplementary analyses were conducted to evaluate the robustness and interpretability of the primary findings.

S5.1 Alternative temporal control specifications

To assess whether the main findings were sensitive to the temporal adjustment strategy, we refitted the models using alternative temporal control structures, including month fixed effects, year fixed effects, combined year-month fixed effects, and year-quarter fixed effects.

Across these specifications, the inverse association between vapour pressure deficit (VPD) and childhood acute respiratory symptoms (ARS) in India's arid macroclimate remained directionally consistent in both single-exposure and bivariable models. Temperature associations in the same stratum were also generally inverse in single-exposure models but were less consistent in bivariable

models. In contrast, findings in other country-macroclimate strata varied more in direction and statistical support across specifications. These analyses supported the temporal robustness of the main VPD finding in India's arid macroclimate while reinforcing cautious interpretation in smaller or less consistent strata.

S5.2 Probability-weighted analyses in India

As a sensitivity analysis to the DHS complex sampling design, we conducted individual-level probability-weighted analyses in India, the largest contributing country. Sampling weights were defined as v005/1e6 and normalised to have mean 1 within each fitted model. Weighted mixed-effects logistic regression models with a random intercept for survey cluster were attempted; when these models were unstable, weighted logistic regression models with cluster-robust standard errors were used as a fallback. These models were interpreted as sensitivity analyses and were not directly equivalent to the primary cluster-month models.

S5.3 Bivariable models including temperature and VPD

To examine whether observed associations were sensitive to inclusion of correlated meteorological indicators, secondary bivariable models including both temperature and VPD were fitted within each country-macroclimate stratum.

S5.4 Alternative handling of solid-fuel data

In the primary analyses, household solid-fuel information was aggregated to the cluster-month level as the proportion of households using solid fuels among those with available data. As a sensitivity analysis, we evaluated alternative handling of missing fuel data using a missing-indicator approach.

S5.5 Exposure timing

To assess sensitivity to exposure timing, associations for contemporaneous monthly exposure (lag 0) were compared with one-month lagged exposure (lag 1) where data permitted.

S5.6 Random-effects assessment

Mixed-effects models were compared with fixed-effects logistic regression models using differences in Akaike Information Criterion (ΔAIC), estimated random-intercept variance, and approximate likelihood ratio tests, acknowledging boundary constraints in variance-component testing.

S5.7 Socioeconomic interaction

To explore whether the association between VPD and ARS varied across socioeconomic context, an interaction term between VPD (z score) and household wealth (z score) was evaluated in supplementary models.

S5.8 PM2.5 exploratory analyses

To explore whether the observed VPD-ARS association in India's arid macroclimate might reflect co-variation with ambient particulate air pollution, we examined satellite-derived monthly PM2.5 concentrations. Pearson correlations between PM2.5 and meteorological variables were calculated, and linear regression models with PM2.5 as the outcome were fitted as exploratory analyses.

S6. Estimation of adjusted absolute risks

In India's arid macroclimate, adjusted absolute risks and risk differences were estimated using marginal standardisation. Predicted risks were calculated at representative VPD values while holding covariates at their observed distributions. Confidence intervals were derived from simulations based on the fixed-effect covariance matrix. Exploratory restricted cubic spline models (three degrees of freedom) were additionally fitted to assess whether the observed association departed materially from linearity over the exposure range.

Supplementary Results

Table S1. Exact primary model estimates underlying Figure 2.

Country	Macroclimate	Exposure	OR (95% CI)	p value
Bangladesh	Temperate	Temperature	0.13 (0.04–0.41)	0.0006
Bangladesh	Temperate	VPD	0.88 (0.42–1.84)	0.7280
Bangladesh	Tropical	Temperature	2.88 (0.99–8.40)	0.0525
Bangladesh	Tropical	VPD	1.55 (0.58–4.12)	0.3825

Country	Macroclimate	Exposure	OR (95% CI)	p value
India	Arid	Temperature	0.64 (0.46–0.88)	0.0072
India	Arid	VPD	0.56 (0.44–0.71)	<0.001
India	Temperate	Temperature	0.99 (0.85–1.16)	0.9244
India	Temperate	VPD	0.97 (0.82–1.15)	0.7391
India	Tropical	Temperature	0.55 (0.32–0.97)	0.0397
India	Tropical	VPD	1.04 (0.64–1.67)	0.8868
Nepal	Temperate	Temperature	1.30 (0.78–2.16)	0.3170
Nepal	Temperate	VPD	1.25 (0.79–1.98)	0.3334
Timor-Leste	Tropical	Temperature	NA	NA
Timor-Leste	Tropical	VPD	NA	NA

Odds ratios (ORs), 95% confidence intervals (CIs), and p values are shown for the primary unweighted single-exposure mixed-effects models fitted within each country-macroclimate stratum. Temperature and VPD were analysed in separate models. Bivariable models including both temperature and VPD are reported separately in the Supplementary Appendix. Estimates were not reported for very small strata where model estimation was unreliable.

Table S2. Comparison of fixed-effects and mixed-effects models across country-macroclimate strata.

ISO3	Macroclimate	Cluster-month observations	Random-intercept variance	ΔAIC (GLM − GLMM)	Approx. LRT p value
BGD	Temperate	293	0.568956	5.211158	0.007245175
BGD	Tropical	172	0.239673	-1.23507	0.38179039
IND	Arid	1676	2.14708	319.3483	7.37E-72
IND	Temperate	4576	2.312104	1148.291	3.88E-252
IND	Tropical	1380	2.332573	103.7848	8.22E-25
NPL	Temperate	242	0	-2	0.999999462
TLS	Tropical	297	—	—	—

This table compares fixed-effects logistic regression models (GLM) with mixed-effects logistic regression models including a cluster-level random intercept (GLMM) across country-macroclimate strata. Model performance was evaluated using the estimated variance of the random intercept, differences in Akaike Information Criterion (ΔAIC = AIC_GLM − AIC_GLMM), and approximate likelihood ratio tests (LRT).

Positive ΔAIC values indicate improved model fit with inclusion of a random intercept. Likelihood ratio tests are reported for reference but should be interpreted cautiously due to boundary constraints when testing variance components. Support for cluster-level random intercepts varied across strata and was strongest in the larger Indian strata; in some smaller strata, random-effect variance estimates were zero or model comparison metrics could not be reliably estimated.

Table S3. Probability-weighted individual-level sensitivity analyses in India by macroclimate.

Macroclimate	Model specification	Exposure	OR	95% CI	p-value
Cold	Temperature-only	Temperature (z-score)	1.00	0.64–1.54	0.984
Cold	VPD-only	VPD (z-score)	7.65	1.16–50.39	0.034
Cold	Bivariate (T + VPD)	Temperature (z-score)	0.51	0.22–1.18	0.117
Cold	Bivariate (T + VPD)	VPD (z-score)	33.72	3.26–348.65	0.003
Arid	Temperature-only	Temperature (z-score)	0.43	0.33–0.57	<0.001
Arid	VPD-only	VPD (z-score)	0.56	0.48–0.65	<0.001
Arid	Bivariate (T + VPD)	Temperature (z-score)	0.66	0.44–1.00	0.051
Arid	Bivariate (T + VPD)	VPD (z-score)	0.66	0.50–0.87	0.003
Temperate	Temperature-only	Temperature (z-score)	1.01	0.91–1.12	0.901
Temperate	VPD-only	VPD (z-score)	0.80	0.68–0.93	0.004
Temperate	Bivariate (T + VPD)	Temperature (z-score)	1.21	1.06–1.38	0.004
Temperate	Bivariate (T + VPD)	VPD (z-score)	0.72	0.60–0.87	<0.001
Tropical	Temperature-only	Temperature (z-score)	0.62	0.47–0.82	<0.001
Tropical	VPD-only	VPD (z-score)	0.90	0.70–1.14	0.372
Tropical	Bivariate (T + VPD)	Temperature (z-score)	0.54	0.39–0.75	<0.001
Tropical	Bivariate (T + VPD)	VPD (z-score)	1.07	0.83–1.39	0.587

Models were fitted at the individual level among children younger than 5 years in India, stratified by Köppen–Geiger macroclimate. Sampling weights were defined as v005/1e6 and normalised to have mean 1 within each fitted model. Weighted mixed-effects logistic regression models with a random intercept for survey cluster were attempted; when convergence failed or estimates were unstable within small strata, weighted logistic regression models with cluster-robust standard errors were used as a fallback (fit_type = GLM_ROBUST). All models included fixed effects for survey year and month and adjusted for child sex, age, urbanicity, household wealth, and maternal education. Estimates in the Cold stratum were imprecise with wide confidence intervals and were interpreted cautiously. These weighted individual-level models were fitted as a sensitivity analysis and were not directly equivalent to the primary cluster-month models reported in the main text; they were interpreted primarily in terms of directional robustness.

Table S4. Pairwise correlations between meteorological exposure variables by country.

country	Variable A	Variable B	Pearson r
BGD	AH (z-score)	T (z-score)	0.925658
BGD	T (z-score)	VPD (z-score)	0.674428
BGD	AH (z-score)	VPD (z-score)	0.358822

IND	AH (z-score)	T (z-score)	0.771065
IND	T (z-score)	VPD (z-score)	0.444516
IND	AH (z-score)	VPD (z-score)	-0.1982
NPL	AH (z-score)	T (z-score)	0.933925
NPL	T (z-score)	VPD (z-score)	0.81653
NPL	AH (z-score)	VPD (z-score)	0.672029
TLS	AH (z-score)	T (z-score)	0.802492
TLS	T (z-score)	VPD (z-score)	0.50518
TLS	AH (z-score)	VPD (z-score)	-0.10848

This table reports Pearson correlation coefficients between standardised meteorological exposure variables, including air temperature (T (z-score)), absolute humidity (AH (z-score)), and vapour pressure deficit (VPD (z-score)), calculated separately for each country. The results illustrate substantial correlations between temperature and humidity-related metrics, while correlations involving VPD vary in magnitude and direction across settings, supporting separate modelling of individual exposures to mitigate multicollinearity.

Table S5. Exploratory analyses of PM2.5 and meteorological variables in India's arid macroclimate.

Variable pair	Pearson r
PM2.5 -VPD (z-score)	-0.38
PM2.5 -Temperature (z-score)	-0.64
PM2.5 -Absolute humidity (z-score)	-0.47
VPD (z-score) -Temperature (z-score)	0.61
VPD (z-score) -Absolute humidity (z-score)	-0.08
Temperature (z-score) -Absolute humidity (z-score)	0.73

Pearson correlations were used to assess statistical relationships between ambient PM2.5 concentrations and standardised meteorological variables in India's arid macroclimate. These exploratory analyses indicate that PM2.5 co-varied more strongly with temperature than with VPD in India's arid macroclimate, and that associations of VPD with PM2.5 were sensitive to inclusion of additional moisture metrics, consistent with collinearity among humidity-related variables. Overall, these exploratory findings do not support PM2.5 as a simple proxy for VPD, although confounding by broader seasonal co-variation cannot be excluded.

Checklist S1. STROBE reporting checklist.

Item	Location
Title and abstract	Title; Abstract
Background / rationale	Introduction
Objectives	Introduction, final paragraph
Study design	Methods, Study design and data sources
Setting	Methods, Study design and data sources; Meteorological exposures
Participants / eligibility criteria	Methods, Study population and analytical units

Item	Location
Variables	Methods, Outcome definition; Meteorological exposures; Climate stratification; Covariates
Data sources / measurement	Methods, Study design and data sources; Meteorological exposures; Climate stratification
Bias	Methods, Study population and analytical units; Supplementary and sensitivity analyses; Discussion, limitations paragraph
Study size	Methods, Study population and analytical units; Results, Study population and analytical units
Quantitative variables	Methods, Climate stratification; Covariates; Statistical analysis
Statistical methods	Methods, Statistical analysis
Subgroups and interactions	Methods, Climate stratification; Supplementary and sensitivity analyses
Missing data	Methods, Study population and analytical units; Supplementary and sensitivity analyses
Sampling strategy	Methods, Study design and data sources; Statistical analysis
Sensitivity analyses	Methods, Supplementary and sensitivity analyses; Online Supplementary Document
Participant numbers	Results, Study population and analytical units; Table 1
Descriptive data	Results, Study population and analytical units; Table 1
Outcome data	Results, Study population and analytical units; Table 1
Main results	Results, Country- and macroclimate-specific associations; Figure 2
Risk translation	Results, Absolute risk translation in India's arid macroclimate; Figure 3
Other analyses	Methods, Supplementary and sensitivity analyses; Online Supplementary Document
Key results	Discussion, first paragraph
Limitations	Discussion, limitations paragraph
Interpretation	Discussion
Generalisability	Discussion; Conclusions
Funding	Funding
